# Application of Natural Preservatives for Meat and Meat Products against Food-Borne Pathogens and Spoilage Bacteria: A Review

**DOI:** 10.3390/foods10102418

**Published:** 2021-10-12

**Authors:** Hwan Hee Yu, Young-Wook Chin, Hyun-Dong Paik

**Affiliations:** 1 Department of Food Science and Biotechnology of Animal Resources, Konkuk University, Seoul 05029, Korea; Yuhwanhee@kfri.re.kr; 2 Research Group of Traditional Food, Korea Food Research Institute, Iseo-myeon, Wanju-gun 55365, Korea; ywchin@kfri.re.kr

**Keywords:** natural preservative, food-borne pathogen, meat, food application, antimicrobial

## Abstract

Meat and meat products are excellent sources of nutrients for humans; however, they also provide a favorable environment for microbial growth. To prevent the microbiological contamination of livestock foods, synthetic preservatives, including nitrites, nitrates, and sorbates, have been widely used in the food industry due to their low cost and strong antibacterial activity. Use of synthetic chemical preservatives is recently being considered by customers due to concerns related to negative health issues. Therefore, the demand for natural substances as food preservatives has increased with the use of plant-derived and animal-derived products, and microbial metabolites. These natural preservatives inhibit the growth of spoilage microorganisms or food-borne pathogens by increasing the permeability of microbial cell membranes, interruption of protein synthesis, and cell metabolism. Natural preservatives can extend the shelf-life and inhibit the growth of microorganisms. However, they can also influence food sensory properties, including the flavor, taste, color, texture, and acceptability of food. To increase the applicability of natural preservatives, a number of strategies, including combinations of different preservatives or food preservation methods, such as active packaging systems and encapsulation, have been explored. This review summarizes the current applications of natural preservatives for meat and meat products.

## 1. Introduction

Food-borne pathogens, including *Listeria monocytogenes*, *Staphylococcus aureus*, pathogenic *Escherichia coli*, *Clostridium perfringens*, *Campylobacter* spp., and *Vibrio* spp., cause a large number of illnesses, with substantial damage to human health and economy. According to the World Health Organization (WHO), food contaminated with food-borne pathogens, chemicals, and allergens results in 600 million cases of food-borne illness and approximately four hundred thousand deaths worldwide each year, Moreover, fifty-six million people die every year and approximately 7.7% of people worldwide suffer from foodborne diseases [1,2]. Meat and meat products are essential nutrient sources for humans due to their excellent protein content, essential amino acids, vitamin B groups, and minerals [3]. However, meat and meat products also provide an appropriate environment for spoilage microorganisms or food-borne pathogens due to their high water activity and nutrient factors [4].

The food industry has advanced worldwide, resulting in an enhanced threat of food contamination by pathogenic microorganisms, chemical residues, harmful food additives, and toxins. The multiplication of spoilage and pathogenic microorganisms should be controlled to ensure food safety. Accordingly, food preservation techniques for protecting food from pathogenic bacteria and extending shelf-life include chemical methods, such as the use of preservatives; physical methods, such as heat treatment, drying, freezing, and packaging; and biological methods using microorganisms that have an antagonistic effect on the pathogenic bacteria and produce bacteriocins [5]. Among them, the addition of food preservatives that inhibit the growth of microorganisms is a widely used food protection technique.

Each country has different regulations for food preservatives. In the case of Korea, chemical preservatives including nitrates (below 0.07 g/kg), nitrites (below 0.07 g/kg), and sorbates (below 2.0 g/kg) are allowed for the meat industry [6]. Synthetic preservatives have the advantage for meat processing due to low cost, guaranteed antibacterial effect or shelf-life extending activity, and little effect on taste, flavor, color, and texture. However, synthetic preservatives tend to be less preferred by food consumers because of a number of health concerns regarding their side effects. In previous survey research, food consumers living in Seoul, Korea selected preservatives as the most concerned food additive owing to their negative impacts on health [7]. Sorbic acid, benzoic acid, and their salts have been reported to promote mutagenic and carcinogenic compounds [8,9]. Nitrites and nitrate, used as preservative and coloring agents in meat, have been associated with leukemia, colon cancer, bladder cancer, and others [10,11,12].

Natural preservatives have emerged as alternatives to synthetic preservatives [13]. Natural preservatives have shown potential to provide effective antimicrobial activity while reducing negative health effects. Meat and meat products containing synthetic additives, are a major concern for human health [14]. Hence, meat manufacturers and researchers have begun to consider the use of natural rather than synthetic preservatives. Representatively, the ‘clean label’ food trends, including meat and meat products, began in the UK in the 1990s and possessed an important source of food marketing. It includes consumer-friendly characteristics, such as synthetic additive-free, least processing, a brief list of food ingredients, and the procedure of traditional methods [15]. In particular, the clean label food material market, including natural preservatives, is likely to value of USD 47.50 billion by 2023, mostly owing to growing consumer requests for all-natural products [16]. In Korea, natural preservatives such as nisin, natamycin, ε-polylysine, and grapefruit seed extract are registered, but they are not approved for meat products, or their concentration is not specified [6].

The replacement of synthetic preservatives with natural preservatives has major positive effects and is being accepted by customers. However, food producers also encounter challenges, including a decrease in price competitiveness due to the relatively high price of natural preservatives and a decrease in antibacterial effect due to food ingredients, such as carbohydrates, proteins, and lipids. In the case of plant-derived substances, standardization is problematic because of the influence of country of origin, soil, and harvest seasons. Furthermore, toxicity evaluation or identification of exact compounds for several plant-derived compounds contained in extracts and essential oils have been performed [17]. To solve these problems, various studies have been conducted to optimize the extraction process, combine other antimicrobial substances, apply active packaging, and encapsulate antibacterial substances to improve their utilization [18,19,20,21,22].

This review summarizes the current knowledge about the application of natural preservatives for meat and meat products against food-borne pathogens and spoilage bacteria.

## 2. The Application Technique of Natural Preservatives to Meat and Meat Products

Natural preservatives are manufactured in a variety of formulations including powder formed by drying methods and liquid forms such as essential oils. Natural preservatives are directly added to meat products and extend the shelf-life by inhibiting bacterial growth. In addition, it is possible to increase the antibacterial effect of natural preservatives through a combination of other food processing methods.

In the case of plant-derived natural preservatives, it is necessary to consider the form applied to food [17]. They are commonly prepared in the form of extracts using organic solvents, water, and essential oils. The plant extracts obtained from rosemary, chestnut, sage, cranberry, oregano, grape seed, and others have been used as meat preservatives. Many studies have been conducted to apply plant-derived substances to meat products in the form of essential oil because the antibacterial effect of essential oil type is better than that of extract type. However, it is difficult to apply large amounts of essential oil to food because of its distinct organoleptic properties. Recent developments have attempted to solve this problem by applying essential oils with other antibacterial substances. The advantage of this application is that it reduces the amounts of essential oils with strong flavor and increases antioxidant and antibacterial effects through synergistic effects. In terms of industrial perspective, if synthetic preservatives cannot be completely replaced with natural preservatives, due to industrial problems, such as increasing economic costs or the complexity of the product manufacturing process, they could be replaced gradually by composing a mixed formulation of synthetic preservatives and natural preservatives [23,24,25,26].

The gamma irradiation and high-pressure processing (HPP) treatment are physical food-processing methods that can further increase the antibacterial efficacy of natural preservatives. Unlike thermal food processing, these two food processing techniques could be used for pasteurization of raw meat because it has a minor effect on food composition. In 1997, the WHO, the Food and Agricultural Organization (FAO), and the International Atomic Energy Agency (IAEA) concluded that foods processed in proper doses of irradiation are nutritionally sufficient and safe to consume [27,28]. Currently, irradiation is permitted for food preservation in more than sixty countries [29]. Recent approaches in food irradiation have involved the use of combined treatments with natural preservatives to reduce irradiation doses. In previous studies, gamma irradiation of medium doses (2–6 kGy) with natural compounds and active packaging has been applied to extend the shelf-life of meat and meat products [30,31].

HPP is also a non-thermal technique for food preservation that inhibits the growth of microorganisms and maintains the natural properties of food. Generally, HPP is performed under high pressures (100–800 MPa) at mild temperature or weak heating [32]. Previous studies have reported the potential capability of combining HPP and natural preservatives including essential oil and antibacterial peptides in alleviating both the processing conditions of HPP and the concentration of natural preservatives while maintaining antibacterial effects [33,34].

Encapsulation is one of the effective approaches for expanding the applicability of natural preservatives to food. The encapsulation was performed with GRAS (generally recognized as safe) materials such as alginate, chitosan, starch, dextrin, and proteins using various techniques including spray-drying, extrusion, freeze-drying, coacervation, and emulsification [35]. The application of natural preservatives to meat is limited due to their characteristics, such as low solubility and bioavailability, rapid release, and easy degradation. Moreover, environmental conditions, such as pH, storage temperature and time, oxygen and light exposures could influence the efficacy of natural preservatives [36]. Through encapsulation, natural preservatives, especially hydrophobic compounds (e.g., essential oil), could improve its stability and expand the versatility of food processing while maintaining the antibacterial effect [37].

Active packaging is an innovative packaging technology that allows for an interaction with the product and its environment to extend shelf-life and to ensure its microbial safety while keeping the original properties of the packaged food [38]. According to the European Union Guidance to the Commission Regulation (EUGCR), active packaging is a type of food packaging with a further beneficial function, while providing a protective barrier against external influence [39]. In the meat industry, antimicrobial active packaging could be applied in several methods which are the incorporation of natural preservatives into a sachet inside the packaging, the packaging film composition with natural preservatives, packaging coated with natural preservatives onto the surface of food, and use of antimicrobial polymers as packaging materials [38].

In the application of microorganism-derived natural preservatives, known as bio-preservation, in which useful microorganisms or their antibacterial substances have antagonistic effects on pathogenic or spoilage microorganisms, are used is also a meat preservation method in the spotlight. This method is mainly involved in lactic acid bacteria, *Lactobacillus* spp., *Leuconostoc* spp., *Pediococcus* spp., and *Lactococcus* spp., that have a GRAS status, widely participate in fermentation processes, and produce various antibacterial metabolites such as organic acids, hydrogen peroxide, and bacteriocins [40]. In terms of the application to meat products, bio-preservation methods included direct inoculation with lactic acid bacteria, which has an inhibitory effect on spoilage or pathogenic bacteria, the inclusion of bacterial strains producing antimicrobial substances in the fermentation starter, and treatment with purified bacteriocins [41].

## 3. Natural Preservatives from Plants and Their Application for Meat and Meat Products

The antibacterial effect of plant-derived natural preservatives is closely related to polyphenols, phenolics, and flavonoids. Plant-derived polyphenols have various classifications and structures, as follows: phenolic acids (caffeic acid, rosmarinic acid, gallic acid, ellagic acid, cinnamic acid), flavones (luteolin, apigenin, chrysoeriol), flavanols (catechin, epicatechin, epigallocatechin, gallocatechin, and their gallate derivatives), flavanones (hesperidin, hesperetin, heridictyol, naringenin), flavonols (quercetin, kaempferol, myricetin), isoflavones (geinstein, daidzin, formononetin), coumarins (coumarin, warfarin, 7-hydroxycourmarin), anthocyanins (pelagonidin, delphinidin, cyanidin, malvidin), quinones (naphthoquinones, hypericin), alkaloids (caffeine, berberine, harmane), and terpenoids (menthol, thymol, lycopene, capsaicin, linalool).

Polyphenols have been recognized for their effective antimicrobial properties. Although the antimicrobial mechanism has not yet been clearly elucidated, previous studies have reported the following [10,42]: (1) cell membrane-disturbing molecules, such as the hydroxy group (OH-), which induces the leakage of intracellular components, inactivation of metabolic enzymes, and extinction of the adenosine triphosphate (ATP) structure; (2) direct pH change in the environment by the improvement in proton concentration, reduction of the intracellular pH by separation of acid molecules, and modification of the bacterial membrane permeability; (3) an organic acid in the plant extracts may influence the oxidation of nicotinamide adenine dinucleotide (NADH), thereby eliminating the reducing agent used in the electron transport system.

Table 1 presents the application of plant-derived preservatives for the inhibition of spoilage bacteria or food-borne pathogens in meat and meat products.

### 3.1. Rosemary

Rosemary (*Rosmarinus officinalis* L.) is a perennial herb with woody, aromatic, and evergreen needle-like leaves. Originally from the Mediterranean region, it is broadly distributed throughout the globe. Rosemary has been used as a spice and flavoring agent in food [65]. Rosemary essential oil is known to contain approximately fifteen kinds of bioactive compounds [66,67]. The principal compound was 1,8-cineole (35.32%). Other major compounds were camphor, α-pinene, trans-caryophyllene, α-thujone, and borneol.

Soyer et al. [43] reported the antibacterial effect of rosemary ethanol extracts against *L. monocytogenes* in beef. The application of 45% rosemary ethanol extract for *L. monocytogenes* on beef led to a 2 log colony-forming unit (CFU)/g reduction in the incubation at 4 °C for 9 d.

In chicken meat, the effect of rosemary essential oil on the inhibition of *Salmonella* Enteritidis and spoilage protective effects at 4 and 18 °C was investigated [44]. The 5 mg/mL of rosemary essential oil induced the decrease in coliform, aerobic bacteria, lactic acid bacteria, and anaerobic bacteria at 18 °C for 24 h. Compared with the untreated chicken meat, the reductions of 1.75 log CFU/g (coliform), 0.87 log CFU/g (aerobic bacteria), 1.05 log CFU/g (lactic acid bacteria) and 1.28 log CFU/g (anaerobic bacteria) were observed in the group treated with rosemary essential oil at 18 °C. Rosemary oil reduced *S*. Enteritidis by more than 2 log CFU/g at 18 °C, but less than 1 log CFU/g at 4 °C.

The rosemary essential oil applied with modified atmosphere packaging for the inhibition of food-borne pathogens (*S*. Typhimurium and *L. monocytogenes*) in poultry filets under refrigerated conditions for 7 d was investigated [45]. The 0.2% rosemary essential oil did not affect the sensory profile and inhibited the growth of both pathogens in laboratory media within 24 h. Treatment with 0.2% rosemary essential oil did not affect the reduction in *S*. Typhimurium, but showed weak antibacterial activity against *L. monocytogenes* until the first day of storage (approximately 0.1 log CFU/g reduction compared to control).

### 3.2. Sage

Sage (*Salvia officinalis* L.), belonging to the Lamiaceae family, has been used since prehistoric eras because of its flavor, taste, therapeutic, and preservative properties. Sage is known to contain considerable amounts of rosemary acid, *p*-coumaric acid, and benzoic acid. Its essential oils, camphor, carvacrol, R(+) limonene, and linalool are the major components in terms of content [46].

Cegiełka et al. [46] reported that the antibacterial effects of various sage preparations were assessed for low-pressure mechanically separated meat (MSM) in vacuum packaging stored at −18 °C for 9 months. MSM from chickens with the addition of sage extracts inhibited the growth of all groups of microorganisms (mesophilic aerobic bacteria, psychrotrophic bacteria, *Enterobacteriaceae*, coliforms, and enterococci). The most effective antibacterial effect was exhibited by the 0.1% sage essential oil-treated groups.

The antibacterial effect of sage essential oil (0.625%) on the survival of *L. monocytogenes* in Sous-vide cook-chill beef stored in refrigerated storage (2 or 8 °C) for 28 d [47]. A decrease of 1 log CFU/g of *L. monocytogenes* was detected in the sage essential oil-treated groups compared to the control at 2 °C. Although exponential growth was observed from day 14, lower *L. monocytogenes* counts of approximately 1 log CFU/g were detected in sage essential oil-treated samples stored at 8 °C.

### 3.3. Thyme

Thyme (*Thymus vulgaris*) is a representative herb used together with meat and meat products. The application of thyme in meat products can elevate antioxidant, antibacterial, shelf-life extension, and sensory properties.

In meat sausage, thyme essential oil inhibited 2.69 log CFU/g of coagulase-positive *Staphylococcus* and 4.41 log CFU/g of aerobic mesophilic bacteria, respectively, at a concentration of 0.95% by mixing with 1% (*w*/*w*) powdered beet juice. Moreover, the sensory properties, odor, flavor, and overall acceptability improved [48]. 

The 1% thyme oil led to the reduction in *S. enterica* by 3 log CFU/g during the margination process with lemon juice and 0.5% *Yucca schidigera* extract in raw chicken breast [49]. The major composition of the thyme oil revealed 51.1% and 24.1% thymol and O-cymene, respectively. The antibacterial effects of thyme may be due to additive or synergistic effects with its major and/or minor components. Thymol and its synergistic effect with other phenolic compounds, such as carvacrol, *p*-cymene, and γ-terpinene, can change the permeability of the bacterial cell wall, leading to cell death [68].

Thyme essential oil encapsulated with casein and maltodextrin was evaluated for its antibacterial potential in vitro and in situ (hamburger-like meat products) [50]. The encapsulated thyme essential oil showed the same minimum inhibitory concentration (0.1 mg/mL) against *E. coli*, *S.* Typhimurium, *S. aureus*, and *L. monocytogenes* as that of the unencapsulated thyme essential. In the treated groups with 1% (*v*/*v*) of encapsulated thyme essential oil for meat, the *E. coli* counts were decreased from 23 most probable number (MPN)/g to 0 MPN/g, which was similar to the conventional preservative (sodium nitrate) used as a control until 14 d of refrigerated storage (4 °C).

### 3.4. Oregano

Oregano (*Origanum vulgare*) is regularly used in Mediterranean foods. The oregano essential oil has recognized antibacterial and antioxidant properties for the extension of shelf-life. The antibacterial effects of oregano were due to two bioactive polyphenols, thymol and carvacrol [69].

The component of oregano essential oil and its impact on the shelf-life of black wildebeest *Biceps femoris* muscles was investigated at 2.6 °C [51]. The components of oregano oil were thymol, carvacrol, ρ-cymene, β-caryophyllene, γ-terpinene, α-humulene, and α-pinene; among them, carvacrol (42.94%) and thymol (17.40%) were the highest. The total viable counts and lactic acid bacteria reached the spoilage limit (7 log CFU/g) after 3 d. The growth rates for total viable counts and lactic acid bacteria in the treated group were 40% higher than those in the untreated groups.

The combinatorial effect of oregano essential oil with caprylic acid was studied in vacuum-packed minced beef [52]. The addition of 0.2% oregano essential oil with 0.5% caprylic acid and 0.1% citric acid in minced beef reduced the counts of lactic acid bacteria by 1.5 log CFU/g in vacuum packaging. Moreover, the cell counts of psychrotrophic bacteria and *L. monocytogenes* were reduced by more than 2.5 log CFU/g at 3 °C for 10 d.

Oregano essential oil inhibits the growth of bacteria by releasing volatile components during the drying process. It was reported that the addition of oregano essential oil composed of carvacrol (64.5%), *p*-cymene (5.2%), and thymol (2.9%) inhibited *S*. Enteritidis and *E. coli* in the beef drying process [53]. For drying, a filter paper was soaked with oregano essential oil and placed in front of the fan of the drier. The beef samples were dried at 55 °C for 6 h. Consequently, both bacteria (*S*. Enteritidis and *E. coli*) were not detected after treatment with 3 mL of oregano essential oil.

### 3.5. Chestnut

*Castanea crenata* was classified into the *Castanea* family and is a woody plant native to East Asia, including Korea and Japan. *Castanea sativa* is one of the most important Castanea families and food resources of European areas for long periods. Chestnut shells contain abundant phenols and hydrolyzable tannins [70].

Lee et al. [54] reported that chestnut inner shell extracts using ethanol exhibited antimicrobial effects against *C. jejuni* in chicken meat at a concentration of 2 mg/mL. The polyphenol and flavonoid contents of chestnut inner shell ethanol extracts were 532.96 ± 3.75 mg gallic acid/100 g and 12.28 ± 0.03 mg quercetin/100 g, respectively. 

Zamuz et al. [55] investigated the influence of chestnut extracts (*Castanea sativa*) on the leaf, bur, and hull of beef patties under refrigerated conditions (2 ± 1 °C) for 18 d to extend shelf-life. Among the chestnut extracts from leaf, bur, and hull, only the leaf extract at a concentration of 1000 mg/kg had weak antimicrobial activity. The lactic acid bacteria and *Pseudomonas* spp. were reduced by 0.37 log CFU/g and 0.33 log CFU/g at 7 d, respectively.

### 3.6. Grapefruit Seed Extract (GSE)

GSE is a by-product of *Citrus paradise*. GSE contains various phenolic compounds and flavonoids, such as catechin, citric acid, naringenin, procyanidin, and epicatechin gallate. GSE has been described to have wide-ranging spectrum antimicrobial, antiparasitic, and antifungal activities [71]. Polyphenols in GSE are unstable but can be chemically modified to become more stable using quaternary ammonium compounds, such as benzethonium chloride, during the industrial procedure of commercial GSE preparations [72]. 

Juneja et al. [56] reported the bacteriostatic effect of commercial GSE (Citricidal^®^) on sous-vide chicken products against *C. perfringens*. The cell numbers of *C. perfringens* were consistently approximately 2.5 log CFU/g regardless of the treatment or control groups until 9.5 h of stored at 19 °C; however, the storage of the control and 50 or 100 ppm GSE treated groups at 25 °C for more than 6 h resulted in fast growth rates of *C. perfringens*, showing 2–3 log CFU/g. GSE concentrations at 200 ppm inhibited the growth of *C. perfringens* stored at 19 and 25 °C.

The active packaging system for the inhibition of food-borne pathogens used mixed natural preservatives consisting of GSE (80 mg/m^2^) with cinnamaldehyde (200 mg/m^2^) and nisin (60 mg/m^2^) was assessed for beef storage [57]. Active packaging showed lower counts of psychrotrophic and anaerobic bacteria compared to the control groups at 1–2 log CFU/g. The packaged beef samples with mixed natural preservatives showed a decrease in *L. monocytogenes*, *S. aureus*, and *C. jejuni* for approximately 4.7 log CFU/g, 0.81 log CFU/g, and 3.1 log CFU/g compared to wrapped packaging at 28 d of refrigerated storage, respectively. In particular, *C. jejuni* was observed below the detection limit after 21 d of storage.

### 3.7. Cinnamon

Cinnamon is a native plant in Asia that is acquired from the inner bark of the genus *Cinnamomum*. Cinnamon contains several active compounds, such as cinnamaldehyde, eugenol, cinnamyl acetate, L-borneol, β-caryophyllene, caryophyllene oxide, camphor, L-bornyl acetate, α-terpineol, α-cubebene, α-thujene, and terpinolene [73].

Khaleque et al. [58] reported that cinnamon (*Cinnamomum cassia*) essential oils could inhibit *L. monocytogenes* in ground beef at refrigerated (0 and 8 °C) and frozen (–18 °C) conditions. The concentration of 5.0% cinnamon essential oil to decrease by 3.5–4.0 log CFU/g of *L. monocytogenes* at 0 and 8 °C for 7 d. Under frozen conditions, *L. monocytogenes* was reduced by 3.5–4.0 log CFU/g over 60 d.

The antibacterial effect and shelf-life extending activity were evaluated using a chitosan edible coating containing 0.6% cinnamon essential oil on roast duck slices under modified atmosphere packaging (30% carbon dioxide (CO_2_)/70% nitrogen (N_2_)) at storage at 2 ± 2 °C for 21 d [59]. The edible coating with cinnamon essential oil showed total viable counts reduced by 1 log CFU/g compared to the control after 14 d of storage. This was similar to the results of *Enterobacteriaceae* counts. The number of lactic acid bacteria was lower than that of the control until the day 7 of storage, but there was no significant difference from day 11 of storage. Notably, the growth of *Vibrio* spp. was delayed using edible coating with cinnamon essential oil within the earlier period of storage as a result of microbial diversity sequencing.

### 3.8. Turmeric

Turmeric (*Curcuma longa* L.) has long been used as a flavor and color agent in food and traditional medicine to treat various diseases, mainly in South and East Asia [74]. The main active compounds of turmeric originate from its constituents, called curcuminoids. Curcuminoids (curcumin, demethoxycurcumin, and bis-demethoxycurcumin) content of turmeric varies between about 2–9% based on its growth environments, such as cultivar, soil, and climatic conditions [75].

The antibacterial effect of turmeric on chicken breast meat was assessed for *E. coli* and *S. aureus* stored at 4 °C for 48 h [60]. When 1% turmeric powder was added, no difference in *S. aureus* counts was observed between the turmeric treated and control groups. In the case of *E. coli*, a reduction of 0.2 log CFU/g was observed, but this was not statistically significant.

In another study, chicken meat was treated with turmeric powder and gamma irradiation to improve meat quality and stability [61]. The total aerobic bacteria and coliforms were completely decontaminated with 3% turmeric powder and 2 kGy of gamma irradiation at 4 °C for 14 d.

The microbial characteristics of edible coatings using turmeric starch and bovine gelatin were investigated in frankfurter sausages [62]. The edible coating was developed with a 5% (*w*/*w*) aqueous solution of turmeric starch and gelatin. The microbial growth of the coated sausages stored at 5 °C for 20 d decreased by 2.21, 1.01, and 1.65 log CFU/g for mesophilic bacteria, lactic acid bacteria, and psychotropic bacteria, respectively. At 10 °C, the decreases were 1.57, 2.14, and 1.99 log CFU/g for mesophilic bacteria, lactic acid bacteria, and psychotropic bacteria, respectively.

### 3.9. Plant-Derived Antimicrobial Peptides (AMPs)

Plant-derived AMPs have been studied for their potential to inhibit different pathogens, including food spoilage microorganisms, food poisoning bacteria, mold, and yeast species [76]. 

The antibacterial peptide Leg1 from chickpea legumin has been reported in the meat application of plant-derived AMPs [63]. Raw pork was pretreated with Leg1 and inoculated with *E. coli* and *B. subtilis*. The bactericidal activity was measured at 37 °C for 16 h. The minimum bactericidal concentrations of Leg1 on pork meat were 125 µM and 15.6 µM for *E. coli* and *B. subtilis*, respectively. This was the same concentration as the MBC of nisin, bacteriocin from *Lactococcus lactis*, for the tested strains.

The AMPs from pea (11SGP) and red kidney bean (RBAH) were used to extend the shelf-life of raw buffalo meat [64]. In laboratory media, the Gram-positive (*L. monocytogenes*, *B. cereus*, *and Streptococcus pyogenes*) and Gram-negative (*E. coli*, *Pseudomonas aeruginosa*, *Acinetobacter baumannii*) bacteria were inhibited by 11GSP (60 µg/mL) and Gram-negative bacteria by 60% and Gram-positive bacteria by 90%. RBAH (60 µg/mL) alleviated the growth of Gram-negative bacteria by 56% and Gram-positive bacteria by 85%. In buffalo meat, the counts of mesophilic bacteria of 11SGP (400 µg/g) and RBAG (400 µg/g) treated groups decreased by 1.60 log CFU/g and 1.94 log CFU/g compared to the control groups. For psychrophilic bacteria, 11SGP and RBAG reduced by 1.10 log CFU/g and 1.47 log CFU/g, respectively, after 15 d of refrigerated storage (4 °C).

## 4. Natural Preservatives from Animals and Their Application for Meat and Meat Products

Various antibacterial systems of animal sources are associated with defense mechanisms against external intruders. The preservatives derived from animal sources include lysozymes, lactoferrin, ovotransferrin, lactoperoxidase, AMPs from livestock animals, and polysaccharides.

Lysozyme can suppress several Gram-positive bacteria because of its distinctive ability to injure bacterial membranes by hydrolyzing the 1,4-β-linkage between N-acetyl-D-glucosamine and N-acetyl-muramic acid of peptidoglycan in the bacterial membrane [10,77]. Peptide-based antibacterial substances containing AMPs from animal sources, ovotransferrin, and lactoferrin could influence cell membranes or synthesize ATP, peptides, and enzymes. The antibacterial mechanism of AMP has been reported to attach to the bacterial cell membrane and disturb its integrity, resulting in cell lysis. AMPs may also exert more complex activities that inhibit metabolic and translational systems [78]. The ovotransferrin isolated from eggs increased the cell membrane permeabilization of Gram-positive and Gram-negative bacteria. Moreover, ovotransferrin destroyed cell membrane integrity, increased the permeability of pathogen membranes, and induced morphological changes. Lactoferrin has antibacterial effects related to the large cationic patches present on the surface and iron impoverishment [79]. Lactoferrin has an antibacterial effect only when in its iron-free state and iron-saturated lactoferrin has a limited antimicrobial activity [80]. Lacroperoxidase oxidizes the sulfhydryl groups of proteins present in the bacterial membrane, which could be injured by the efflux of potassium ions, amino acids, peptides, and enzymes [81].

Table 2 presents the application of animal-derived preservatives for the inhibition of spoilage bacteria or food-borne pathogens in meat and meat products.

### 4.1. Lysozyme

Lysozyme (muramidase or N-acetyl-muramichydrolase) is mainly extracted from hen egg whites and is known as an antimicrobial enzyme. Lysozyme is a glycoside hydrolase that hydrolyses the linkages in peptidoglycan at Gram-positive bacterial cell wall. It is composed of 129 amino acids, which contain disulfide bonds and tryptophan, tyrosine, and phenylalanine residues [92]. Lysozyme has been used commercially, named Inovapure^®^, against spoilage microorganisms and food-borne pathogens to prolong the shelf-life of raw and processed meat [10]. 

Modified lysozyme, high hydrophobicity, and low hydrolytic activity compared to the lysozyme monomer, at concentrations of 5%, exhibited low microbial growth rates (total viable count 4.59 log CFU/cm^2^; molds and yeasts 2.17 log CFU/cm^2^) in the pork meat surface with modified atmosphere packaging with composites of 50% O_2_, 40% CO_2_, and 10% N_2_ [82].

According to a previous study [83], mixed antimicrobials consisting of lysozyme (250 ppm), nisin (250 ppm), and disodium ethylenediaminetetraacetic acid (EDTA) (20 mM) had antibacterial effects against *L. monocytogenes*, total viable counts, *Enterobacteriaceae*, *Pseudomonas* spp., and lactic acid bacteria in ostrich meat patties with air and vacuum packaging. The mixed lysozyme preparations reduced *L. monocytogenes* below the official detection limit of the EU (<2 log CFU/g) in ostrich meat patties. The treated samples showed a decrease in total viable counts by 1 log CFU/g after 2 d of storage and tended to increase thereafter. *Enterobacteriaceae* and *Pseudomonas* spp. were not affected by the mixed antimicrobials in either packaging atmosphere, and the reduction in lactic acid bacteria was detected at 2 log CFU/g. 

The combination of lysozyme with chitooligosaccharide presented a more effective antibacterial effect against Gram-negative bacteria than lysozyme alone. In minced lamb meat, the mixture of lysozyme and chitooligosaccharide led to complete removal of 3–4 log CFU/g of inoculated *E. coli*, *Pseudomonas fluorescens*, and *B. cereus* during 4 h at ambient temperature. However, *S. aureus* was not completely eliminated, but was reduced up to 2 log CFU/g [84].

### 4.2. Ovotransferrin

Egg white contains 13% ovotransferrin (conalbumin), which is a monomeric 77.9 kDa glycoprotein comprised of 686 amino acid residues. It contains N- and C- globular parts, each of which can reversibly Fe^3+^ and CO_3_^2−^ [93]. Several studies have reported that ovotransferrin is the main constituent of the egg’s defense system for microorganisms, as it renders iron unusable for microbial growth within the albumen [85,94]. 

Seol et al. [85] investigated the antimicrobial effects of ovotransferrin against *E. coli* in fresh chicken breast involved in κ-carrageenan film. The growth of *E. coli* in fresh chicken breast wrapped with active film was 2.7 log CFU/g by the addition of 25 mg of ovotransferrin in combination with 5 mM EDTA. 

In ham models, 25 mg/mL of ovotransferrin with 100 mM sodium bicarbonate (NaHCO_3_) did not show any antibacterial effects against *E. coli* O157:H7 and *L. monocytogenes* in commercial hams, whereas 25 mg/mL ovotransferrin with 0.5% citric acid had bacteriostatic effects against *L. monocytogenes* [86]. 

### 4.3. Lactoferrin

Lactoferrin, a glycoprotein that belongs to the transferrin protein family in milk and milk products as well as neutrophil granules and exocrine secretions in mammals, was able to bind iron within the cells [95]. The ability of this 80 kDa protein to control free iron levels contributes to its bacteriostatic and health-beneficial characteristics, such as stimulating bone growth, protecting the intestinal epithelium, and promoting the immune system in animals [43]. 

In ground beef, application of active lactoferrin, immobilized lactoferrin with glycosaminoglycans, and solubilized in citrate/bicarbonate buffer systems at concentrations of 3% and 5% resulted in 2 log CFU/g reductions of *E. coli* O157:H7 at 10 °C for 9 d. The reduction of *S*. Enteritidis growth was 0.8 log CFU/g when the active lactoferrin concentration was increased to 2.5%. A single application of 0.5% active lactoferrin reduced *L. monocytogenes* in beef, resulting in 2 log CFU/g [43]. 

Bovine lactoferrin (0.5 mg) was tested against *E. coli* O157:H7 and *P. fluorescens* inoculated on chicken with HPP treatments between 200 and 500 MPa for 10 min at 10 °C [87]. As a result, *P. fluorescens* was decreased when lactoferrin was combined with HPP treatment at 300 MPa for 2.3 log CFU/g additional reduction compared to only 300 MPa treatment on day 9. Additional reductions in *E. coli* O157:H7 counts obtained by combined treatments remained below 0.5 log CFU/g.

### 4.4. Lactoperoxidase

Lactoperoxidase is a member of the peroxidase family. It is a ubiquitous active enzyme in bovine milk, which has antimicrobial effects. Bovine lactoperoxidase is a glycoprotein that contains a peptide chain of 78.4 kDa and catalyzes the oxidation of thiocyanate ions (SCN-) in lactoperoxidase, producing oxidizing products, such as hypothiocyanite and hypothiocyanous acid [96]. 

According to Yousefi et al. [81], lactoperoxidase coated with alginate at concentrations of 2, 4, and 6% on the shelf-life of chicken breast filets. The chicken samples with active coating of alginate and 6% lactoperoxidase showed a reduction of *Enterobacteriaceae*, *P. aeruginosa*, and aerobic mesophilic bacteria by approximately 5 log CFU/g, 4 log CFU/g, and 2.5 log CFU/g at 16 d of refrigerated storage, respectively.

The antimicrobial effects of lactoperoxidase were also assessed against *L. monocytogenes* and *S*. Enteritidis in sliced dry-cured-ham for 60 d at 8 °C treated with HPP at 450 MPa [88]. The synergistic effect of lactoperoxidase and pressure was confirmed as *S*. Enteritidis decreased below the detection limit (1 log CFU/g). For *L. monocytogenes*, the synergistic effect reduced cell viability by 0.86 log CFU/g compared with untreated samples at the end of storage.

In beef, the effect of lactoperoxidase on the growth of the inoculated pathogens (4 log CFU/g) composed of *S. aureus*, *L. monocytogenes*, *E. coli* O157:H7, *S*. Typhimurium, *P. aeruginosa*, *Yersinia enterocolitica*, and indigenous microbiota was investigated [89]. All pathogens used in the experiment were reduced compared to the control at a chilling regime (−1 to 12 °C) for 42 d. The total aerobe and *Pseudomonas* spp. increased less in the lactoperoxidase treated group than in the control group, but the antibacterial effect was not exhibited for anaerobes and lactic acid bacteria.

### 4.5. Livestock Animal-Derived AMPs

Livestock animal-derived products have been used as a source of AMPs [97]. Among these by-products of livestock, blood, bones, collagen, gelatin, liver, lungs, placenta, skin, and visceral mass are important sources of AMPs, as well as muscle parts [98]. 

The bovine cruor, a slaughterhouse byproduct containing mainly hemoglobin, broadly described as a rich source of fibrin proteins, was investigated for the extraction of AMPs. The faction named α137–141 (polypeptide with five components, Thr-Ser-Lys-Tyr-Arg), a small (0.65 kDa), and hydrophilic AMPs deviated from hemoglobin. The α137–141 preservative (0.5%, *w*/*w*) had bacteriostatic effects on the total microbial population, coliform bacteria, yeasts, and molds at 4 °C for 14 d on beef [90]. 

The AMPs isolated from porcine leukocytes had antibacterial effects on the proliferation of *S*. *aureus* and *E. coli* inoculated in ground meat (boneless ham) and sausage minces [91]. The 20 μg/g AMPs decreased by 1.3 log CFU/g of *S. aureus* and 1.5 log CFU/g of *E. coli* in ground meat. It was also achieved that 160 μg/g of AMPs had the best inhibition and decreased in 3.9 log CFU/g of *S. aureus* and 3.3 log CFU/g of *E. coli* at 6 h in ground meats. In sausage mince, the AMPs at concentrations of 160 μg/g could decrease by 3 log CFU/g of *S. aureus* and 2.7 log CFU/g of *E. coli* at 12 h. After 24 h of storage, no visible colonies of *S. aureus* or *E. coli* were detected in the sausage mince.

## 5. Natural Preservatives from Microorganism and Their Application for Meat and Meat Products

Lactic acid bacteria (LAB) strains secrete several bacterial growth inhibitory substances (organic acids, diacetyl, phenyl-lactate, hydroxyphenyl-lactate, cyclic dipeptides, hydroxy fatty acid, propionate, and hydrogen peroxide), bacteriocins (nisin, acidophilin, bulgaricin, helveticin, lactacin, pediocin, plantarim, diplococcin, and bifidocin), and bacteriocin-like inhibitory substances (BLIS), which exhibit antibacterial activity and can control spoilage microorganisms and food-borne pathogens [96,99,100]. Among various bacteriocins, commercial bacteriocin preparations have been applied using nisin and pediocin.

Bacteriocins are peptides or proteins with antibacterial and antifungal effects that produce bacteria, mainly lactic acid bacteria. These compounds are considered potential natural preservatives because of their inhibitory effects on food spoilage bacteria or pathogens [10]. LAB bacteriocins vary in accordance with molecular size, chemical structure, modifications during biosynthesis, presence of modified amino acid residues, and antimicrobial mechanisms. Therefore, LAB bacteriocins can be categorized into two major classes: class I (lanthionine-containing antibiotics) with three subclasses (Ia, Ib, and Ic) and class II with four subclasses (IIa, IIb, IIc, and IId) [101]. Class I bacteriocins generally include 19–50 amino acid residues (<5 kDa) and are largely post-translationally modified, ensuring non-standard amino acids, such as lanthionine, β-methyllanthionine, and labyrinthine [102]. Accordingly, class I bacteriocins are further subdivided into class Ia (lantibiotics), class Ib (labyrinthopeptins), and class Ic (sanctibiotics). Class II bacteriocins comprise small, heat-stable, non-modified peptides (<10 kDa). It can be further subdivided into class IIa (pediocin-like bacteriocins), class IIb (non-modified bacteriocins with two or more peptides), class IIc (circular bacteriocins), and class IId (non-pediocin-like bacteriocins) [103]. Pediocin-like bacteriocins (class IIa) can be regarded as the main subgroup among all classified LAB bacteriocins. Class III bacteriocins are classified as high molecular weight (>30 kDa) and thermally unstable peptides. Class IV bacteriocins are large peptides complexed with lipids or carbohydrates [96].

The bacterial cell surface exhibits a negative charge because the anionic characteristics of the cell membrane consist of phosphatidylethanolamine, phosphatidylglycerol, lipopolysaccharide, lipoteichoic acid, and cardiolipin, and is generally captured by the positively charged bacteriocins [103]. The cationic charged groups of bacteriocins electrostatically interact with the anionic bacterial cell surface, while the hydrophobic surfaces are attached to the membrane and traverse the lipid bilayer. The bacteriocins self-associate or polymerize to develop complexes after passing through the lipid bilayer [104]. Finally, bacteriocins induce cell death by increasing the permeability of the bacterial membrane, forming pores that cause dissipation of the proton motive force, exhaustion of ATP, and leakage of intracellular substrates [103,105]. Gram-positive bacteria-derived bacteriocins only perform for Gram-positive bacteria and are not effective against Gram-negative bacteria because of their different membrane compositions and selective membrane permeability [106]. These disadvantages could be compensated by mixing processing with other preservatives and the application of further preservation methods.

Table 3 presents the application of microorganism-derived preservatives for the inhibition of spoilage bacteria or food-borne pathogens in meat and meat products.

### 5.1. Nisin

Nisin is the most representative class I bacteriocin. Nisin is produced by several strains of *Lactococcus lactis*, a species that is widely used for dairy production. It was first approved as a food preservative in the United Kingdom in the 1950s and is now widely used worldwide and is permitted in over 50 countries [119]. The structure of nisin consists of a polypeptide with 34 amino acids, a 3.5 kDa molecular mass, and contains methyllanthionine and lanthionine groups. It has antimicrobial activities against a wide range of Gram-positive bacteria, including *Staphylococcus* spp., *Bacillus* spp., *Listeria* spp., and *Enterococcus spp*. Nisaplin^®^ is a typical commercial nisin formulation. 

Nisin could provide long-lasting bacteriostatic effects on pathogenic microorganisms in beef jerky at room temperature. Lee et al. [107] investigated the shelf-life extensive effect of nisin in *B. cereus* inoculated with beef jerky. In beef jerky without nisin, the counts of mesophilic bacteria and *B. cereus* increasing is unlikely for beef jerky treated with nisin at 25 °C for 60 d. *B. cereus* grew after 3 d in the 100 IU nisin/g treated groups and after 21 d in the 500 IU/g nisin-treated groups. 

The nisin-containing fermentate from *L. lactis* 537 strain was evaluated for the inhibition *of L. monocytogenes* in ready-to-eat (RTE) sliced ham. The addition of the fermentate to RTE sliced ham led to an immediate decrease in *L. monocytogenes* counts from 3 log CFU/g to below the detection limit stored at 4 °C (20 CFU/g) [108].

Nisin with cinnamaldehyde and grapefruit seed extract presented synergistic antibacterial effects [71]. It reduced the counts of *L. monocytogenes* by 3 log CFU/g in raw pork loin at 4 °C for 12 h. The minimum inhibitory concentration of nisin against *L. monocytogenes* was 250 ppm in laboratory media, but it was possible to reduce the concentration of 5–6 ppm against the growth of *L. monocytogenes* by mixing with natural antibacterial substances in pork. 

### 5.2. Pediocin

*Pediococcus* spp., *Pediococcus acidilactici*, and *Pediococcus pentosaceus* are the main pediocin-producing strains. Pediocin was classified into the bacteriocin group class IIa, characterized as small non-modified peptides (<5 kDa) comprising less than 50 amino acids. [120]. Remarkably, pediocin showed antimicrobial activity even at nanomolar concentrations [121]. Food grade pediocin-containing formulations are commercially available and marketed as ALTA 2341 and MicroGARD [122]. Pediocin has been studied for the inhibition *of Listeria* spp. for meat preservation.

The antibacterial activities of pediocin PA-1 in frankfurters and the *P. acidilactici* MCH14, pediocin PA-1 producing strain, in Spanish dry-fermented sausages were assessed against *L. monocytogenes* and *C. perfringens* [109]. In frankfurters treated with 5000 bacteriocin units (BU)/mL of the pediocin PA-1 produced by *P. acidilactici* MCH14, the *L. monocytogenes* was reduced by 2 and 0.6 log CFU/g after storage at 4 °C for 60 d and at 15 °C for 30 d, respectively. *C. perfringens* decreased with 5000 BU/mL of pediocin PA-1 by 2 and 0.8 log CFU/g after storage at 10 °C for 60 d and at 15 °C for 30 d, respectively. The growth of *L. monocytogenes* was inhibited by the pediocin-producing strain, *P. acidilactici* MCH14, in Spanish dry-fermented sausages at 2 log CFU/g compared to the control.

The bacHA-6111-2, pediocin from *P. acidilactici* HA-6111-2, was applied to Portuguese fermented meat sausage (Alheira) with HPP treatment (300 MPa, 5 min, 10 °C) to inhibit *Listeria innocua* [110]. The bacteriostatic effect was verified for high inoculation counts of *L. innocua* at 4 °C for 60 d. For lower inoculated *L. innocua*, antibacterial effect was observed below 2 log CFU/g from day 3 of storage until the end of storage.

Kumar et al. [111] investigated the antibacterial activities of a mixed preparation containing pediocin from *P. pentosaceus* and *Murraya koenigii* (curry tree) berries in a raw goat meat emulsion at 4 °C for 9 d. The *L. innocua* was reduced for 4.1 log CFU/g in the treated samples concentrations at 8.3 mL pediocin/1000 g of meat emulsion with 10% (*v*/*w*) *Murraya koenigii* berries extract at the end of storage. Moreover, total viable count and psychrophilic count were also observed lower in the treated samples, 2.2 log CFU/g and 1.6 log CFU/g, respectively.

### 5.3. Sakacin

Sakacins, a class II bacteriocin, are mainly produced by *Lactobacillus sakei* or *Lactobacillus curvatus* strains. Commercial sakacin products are currently not presented. Compared to nisin and pediocin, sakacins have a relatively narrow antimicrobial spectrum, especially with effective inhibition against *Listeria* species [123]. 

Dortu et al. [112] assessed the antibacterial effect of the sakacin-producing strain, *L. sakei* CWBI-B1365, and *L. curvatus* CWBI-B28, on the fate of *L. monocytogenes* in raw beef and poultry. In refrigerated (5 °C) raw beef, *L. sakei* induced a decrease in the *L. monocytogenes* concentration by 1.5 log CFU/g after 7 d to 2 log CFU/g after 14 d, and below the detection limit at 21 d. The addition of *L. curvatus* reduced *L. monocytogenes* to below the detection limit after 7 d. However, in poultry, the bacteriocin-producing strain did not affect the inhibition of *L. monocytogenes*. It was assumed that the type of meat may have influenced bacteriocin production by LAB.

The antibacterial activity of different bacteriocin preparations using sakacin Q produced by *L. curvatus* ACU-1 on the meat surface was evaluated against *L. innocua* [113]. The freeze-dried reconstituted cell-free supernatant (3200 AU/mL) was effective for the inhibition of *L. innocua* on the meat surface, decreasing its bacterial cell number to the detection limit (<2 log CFU/g) after 2 weeks of storage at 4–5 °C. The adsorption of sakacin Q to meat products, main ingredients, meat proteins, and fat tissues did not affect its antibacterial activity.

### 5.4. Bacteriocin-Like Inhibitory Substance (BLIS)

BLIS are among the antimicrobial substances produced by microorganisms and are not completely categorized in terms of amino acid composition, molecular size, and nucleotide sequence [124].

In RTE pork ham, the antibacterial effects of BLIS produced by *P. pentosaceus* American Type Culture Collection (ATCC) 43200 were assessed and compared with those of commercially available nisin preparations (Nisaplin^®^) [114]. BLIS showed effective antibacterial activity against *Listeria seeligeri* by 0.74 log CFU/g in RTE ham stored at 4 °C after 2 d. However, a slight increase in *L. seeligeri* counts was detected in the BLIS-treated samples from 6 day to the end of storage. Nisaplin^®^ did not present any antibacterial effect for up to 2 d. After 2 d, Nisaplin^®^ started to induce a decrease in *L. seeligeri* counts throughout the refrigerated storage. This might have been due to the higher sensitivity of BLIS to residual proteases compared to nisin, thus weakening its antibacterial effect.

The BLIS-producing LAB strains, *P. acidilactici* KTU05-7, *P. pentosaceus* KTU05-9, and *L. sakei* KTU05-6, were used to ferment the plant (Jerusalem artichoke, *Helianthus tuberosus* L.), and 5% of the fermented products were tested to inhibit the food-borne pathogen at 18 °C for 12 h in ready-to-cook (RTC) minced pork [115]. As a result, the *P. acidilactici* fermented product presented the highest antimicrobial activity compared to the other strains. The counts of *E. coli*, *Enterococcus faecalis*, *S. aureus*, and *Streptococcus* spp. were reduced by 5.53, 4.37, 4.86, and 3.84 log CFU/g, respectively, compared to the control groups, suggesting that the fermented product of the BLIS-producing strains showed an enhanced antibacterial effect. 

Lee et al. [116] reported that BLIS obtained from *Enterococcus faecium* DB1 inhibited the growth and formation of biofilms of *C. perfringens* in chicken meat. The 2.5 mg/mL of DB1 BLIS suppressed the growth of *C. perfringens* by approximately 30%. *C. perfringens* growth was inhibited by 50% at 5 mg/mL DB1 BLIS. Biofilm formation by *C. perfringens* treated with 5 mg/mL DB1 BLIS was radically reduced by approximately 90% at 4 °C for 72 h compared to the control groups. The 2.5 mg/mL of DB1 BLIS also inhibited biofilm formation by *C. perfringens* under the same conditions. BLIS could inhibit the formation of *C. perfringens* biofilms on chicken surfaces due to its antibacterial effect.

### 5.5. Other Microorganism Sources

The mytichitin-CB peptide, which was isolated from the blood lymphocytes of *Mytilus coruscus*, showed antibacterial effects against Gram-positive bacteria and fungi [125]. The current study investigated the mytichitin-CB peptide expressed by *Pichia pastorisi* and applied it to pork preservation [117]. The total viable counts of the treated group with 6 mg/L of mytichitin-CB derived from *P. pastorisi* was reduced by 33% (1–2 log CFU/g) compared to the control group after storage at 4 °C for 5 d. Mytichitin-CB effectively inhibited total bacterial growth during storage compared to the groups treated with 50 mg/L of nisin. Mytichitin-CB at 6 and 12 mg/L suppressed *Staphylococcus* spp. and *Escherichia* spp., respectively, with a reduction of 1–2 log CFU/g, respectively. Moreover, *Listeria* spp. and *Pseudomonas* spp. were not detected during storage, unlike the control and nisin-treated groups.

Hispidalin is a unique AMP derived from the seeds of *Benincasa hispida* and has been shown to exhibit antimicrobial effects against various microorganisms [126]. Meng et al. reported that hispidalin expressed by *P. pastorisi* was used as a preservative for pork meat [118]. Pork meat treated with 100 μg/mL hispidalin showed bacteriostatic effects during the entire refrigerated storage period. The total viable count of pork with 100 μg/mL hispidalin was 1 log CFU/g lower than that of the control group at 4 °C for 7 d.

## 6. Conclusions

Meat and meat products are excellent nutrient sources due to their abundant protein content, essential amino acids, vitamins, and minerals. However, they are susceptible to contamination by food-borne pathogens and various spoilage microorganisms because of their high water activity and nutrient content. Therefore, the application of preservatives is an indispensable element in livestock food industry to prevent food poisoning, delay spoilage, and extend their shelf life. Industrial preservatives, commonly made up of synthetic chemicals, are not demanded by food customers because of negative health concerns. Therefore, natural preservatives derived from plants (rosemary, sage, chestnut, GSE, and tumeric), animals (lysozyme, lactoferrin, lactoferoxidase, ovotransferrin, and others), and microorganisms (organic acids, bacteriocins, and BLIS) have been explored as alternatives to synthetic chemical preservatives. However, the versatility of natural preservatives compared to synthetic preservatives is limited due to the production cost, standardization, insufficient toxicity studies, and negative sensory effects on food. To compensate for these disadvantages, various applications have been studied for their synergistic effect with other natural preservatives with reduced application concentrations compared to single use, the application of physical treatment (gamma irradation, high pressure processing, and drying), encapsulation, and the possibility of packaging materials. This review summarizes various natural preservatives and application methods to inhibit the growth of food-borne pathogens and spoilage bacteria in livestock foods. Natural preservatives are expected to be in high demand due to consumer and industrial requests. Therefore, it is necessary to explore various applications of existing natural preservatives, while continuously searching for novel ones.

## Figures and Tables

**Table 1:** Application of plant-derived preservatives for the inhibition of spoilage bacteria or food-borne pathogens in meat and meat products.

Sources	Forms	Addition Conditions	Meat and Meat Products	Storage Conditions	Target Microorganisms	Antimicrobial Activities	References
Rosemary	Ethanol extract	45%	Beef	4 °C for 9 d	*Listeria monocytogenes*	2 log CFU/g	[43]
	Essential oil	5 mg/mL	Chicken	18 °C for 24 h	*Salmonella* Enteritidis Coliform Total viable counts Lactic acid bacteria Anaerobic bacteria	1 log CFU/g 1.75 log CFU/g 0.87 log CFU/g 1.05 log CFU/g 1.28 log CFU/g	[44]
	Essential oil	0.2% with modified atmosphere packaging	Poultry fillet	4 °C for 1 d	*Listeria monocytogenes*	0.1 log CFU/g	[45]
Sage	Essential oil	0.1%	Mechanically separated chicken meat	−18 °C for 9 months	Total viable counts Psychrotrophic bacteria *Enterobacteriaceae* Coliform *Enterococcus* spp.	0.5 log CFU/g 0.2 log CFU/g 0.9 log CFU/g 1.5 log CFU/g 1.6 log CFU/g	[46]
	Essential oil	0.625%	Sous-vide cook-chill beef	2 °C for 28 d 8 °C for 28 d	*Listeria monocytogenes*	1 log CFU/g 1 log CFU/g	[47]
Thyme	Essential oil	0.95% with 1% powdered beet juice	Meat sausage	4 °C for 15 d	*Staphylococcus* spp. Aerobic mesophilic bacteria	2.69 log CFU/g 4.41 log CFU/g	[48]
	Essential oil	1% with lemon juice and 0.5% *Yucca schidigera* extract	Raw chicken breast	immersed at 22 °C for 8 h	*Salmonella enteritica*	3–4 log CFU/g	[49]
	Essential oil	1% encapculated with casein and maltodextrin	Hamburger-like meat products	4 °C for 14 d	*Escherichia coli*	23 MPN/g	[50]
Oregano	Essential oil	Addition 1%	Black wildebeest muscle	2.6 ± 0.6 °C for 3 d	Total viable counts Lactic acid bacteria	1.4 log CFU/g	[51]
	Essential oil	0.2% with 0.5% caprylic acid and 0.1% of citric acid	Minced beef	3 °C for 10 d	Lactic acid bacteria Psychrotrophic bacteria *Listeria monocytogenes*	1.5 log CFU/g 2.5 log CFU/g 2.5 log CFU/g	[52]
	Essential oil	3 mL for filter paper	Beef	dried at 55 °C for 6 h	*Salmonella* Enteritidis *Escherichia coli*	4.79 log CFU/g 4.68 log CFU/g	[53]
Chestnut	Inner shell extract	1 mg/mL	Chicken	4 °C for 4 d	*Campylobacter jejuni*	3 log CFU/g	[54]
	Leaf extract	1000 mg/kg	Beef patties	2 ± 1 °C for 18 d	Lactic acid bacteria *Pseudomonas* spp.	0.37 log CFU/g 0.33 log CFU/g	[55]
Grapefruit seed extract	Commercial product (Citricidal^®^)	200 ppm	Chicken	19 and 25 °C for 9.5 h	*Clostridium perfringens*	Bacteriostatic effect	[56]
	Commercial product (DF-100)	Active film of GSE (80 mg/m^2^) with cinnamaldehyde (200 mg/m^2^) and nisin (60 mg/m^2^)	Beef	4 °C for 28 d	Psychrotrophic bacteria Anaerobic bacteria *Listeria monocytogenes* *Staphylococcus aureus* *Campylobacter jejuni*	1–2 log CFU/g 1–2 log CFU/g 4.7 log CFU/g 0.81 log CFU/g 3.1 log CFU/g	[57]
Cinnamon	Essential oil	5.0%	Ground beef	–18 °C for 60 d 0 and 8 °C for 7 d	*Listeria monocytogenes*	3.5–4.0 log CFU/g 3.5–4.0 log CFU/g	[58]
	Essential oil	0.6% with chitosan edible coating under modified atmosphere packaging	Roast duck slice	2 ± 2 °C for 14 d 2 ± 2 °C for 14 d 2 ± 2 °C for 7 d	Total viable count *Enterobacteriaceae* Lactic acid bacteria	1 log CFU/g 1 log CFU/g 0.75 log CFU/g	[59]
Turmeric	Powder	1%	Chicken breast meat	4 °C for 48 h	*Escherichia coli*	0.2 log CFU/g	[60]
	Powder	3% with 2 kGy of gamma irradiation	Chicken meat	4 °C for 14 d	Total viable counts Coliform	Bactericidal effect	[61]
	Residue using supercritical fluid extraction and pressurized liquid extraction	5% with edible coating using starch and bovine gelatin	Frankfurter sausage	5 °C for 20 d	Total viable counts Lactic acid bacteria Psychrotrophic bacteria	2.21 log CFU/g 1.01 log CFU/g 1.65 log CFU/g	[62]
Plant-derived antimicrobial peptides	Leg1 from Chickpea legumin	125 µM 15.6 µM	Raw pork	37 °C for 16 h	*Escherichia coli* *Bacillus subtilis*	Bactericidal effect	[63]
	11SGP from Pea	400 µg/g	Raw buffalo meat	4 °C for 15 d	Total viable counts Psychrophilic bacteria	1.60 log CFU/g 1.10 log CFU/g	[64]
	RBAH from Red kidney bean	400 µg/g	Raw buffalo meat	4 °C for 15 d	Total viable counts Psychrophilic bacteria	1.94 log CFU/g 1.47 log CFU/g	[64]

**Table 2:** Application of animal-derived preservatives for the inhibition of spoilage bacteria or food-borne pathogens in meat and meat products.

Sources	Addition Conditions	Meat and Meat Products	Storage Conditions	Target Microorganisms	Antimicrobial Activities	References
Lysozyme	5% with modified atmosphere packaging	Pork meat	4 °C for 28 d	Total viable counts	4.59 log CFU/cm^2^	[82]
	250 ppm with nisin (250 ppm) and EDTA (20 mM) in vacuum packaging	Ostrich meat patties	4 °C for 8 d 4 °C for 8 d 4 °C for 1 d	*Listeria monocytogenes* Lactic acid bacteria Total viable counts	4 log CFU/g 1 log CFU/g 2 log CFU/g	[83]
	Combination with chitooligosaccharide	Lamb meat	ambient temperature for 4 h	*Escherichia coli* *Pseudomonas fluorescens* *Bacillus cereus* *Staphylococcus aureus*	3–4 log CFU/g 3–4 log CFU/g 3–4 log CFU/g 2 log CFU/g	[84]
Ovotransferrin	25 mg with 5 mM EDTA in κ-carrageenan-based film	Chicken breast	5 °C for 7 d	Total viable counts *Escherichia coli*	1.8 log CFU/g 2.7 log CFU/g	[85]
	25 mg/mL of ovotransferrin with 0.5% citric acid	Ham	4 °C for 8 d	*Listeria monocytogenes*	Bacteriostatic effect	[86]
Lactoferrin	3% and 5% 2.5% 0.5%	Ground beef	10 °C for 9 d	*Escherichia coli* O157:H7 *Salmonella* Enteritidis *Listeria monocytogenes*	2 log CFU/g 0.8 log CFU/g 2 log CFU/g	[43]
	0.5 mg/g with high pressure treatments	Chicken fillet	5 °C for 9 d	*Pseudomonas fluorescens* *Escherichia coli* O157:H7	2.3 log CFU/g 0.5 log CFU/g	[87]
Lactoperoxidase	6% with alginate coating	Chicken breast fillets	4 °C for 16 d	*Enterobacteriaceae* *Pseudomonas aeruginosa* Total viable counts	5 log CFU/g 4 log CFU/g 2.5 log CFU/g	[81]
	40 mg/mL with high pressure processing	Dry cured ham	8 °C for 60 d	*Salmonella* Enteritidis *Listeria monocytogenes*	3–4 log CFU/g 0.86 log CFU/g	[88]
	Dipping into antibacterial solution (0.2 mg glucose, 0.1 mg sodium thiocyanate, 1.9 U lactoperoxidase, and 0.38 U glucose oxidase)	Beef	chilling regime (−1 to 12 °C) for 42 d	*Staphylococcus aureus* *Salmonella* Typhimurium *Listeria monocytogenes* *Escherichia coli* O157:H7 *Pseudomonas aeruginosa* *Yersinia enterocolitica*	1.7 log CFU/g 1.6 log CFU/g 1.8 log CFU/g 0.2 log CFU/g 0.9 log CFU/g 3.9 log CFU/g	[89]
Livestock animal-derived antimicrobial peptide	0.5% of α137–141 from bovine cruor	Beef	4 °C for 14 d	Total viable counts Coliform	Bacteriostatic effect	[90]
	160 μg/g of AMPs isolated porcine leukocyte	Boneless ham	15 °C for 6 h	*Staphylococcus aureus* *Escherichia coli*	3.9 log CFU/g 3.3 log CFU/g	[91]
	160 μg/g of AMPs isolated porcine leukocyte	Sausage mince	15 °C for 24 h	*Staphylococcus aureus* *Escherichia coli*	Bactericidal effect	[91]

**Table 3:** Application of microorganism-derived preservatives for the inhibition of spoilage bacteria or food-borne pathogens in meat and meat products.

Souces	Addition Condtions	Meat and Meat Products	Storage Conditions	Target Microorganisms	Antimicrobial Activities	References
Nisin	100 IU/g 500 IU/g	Beef jerky	25 °C for 3 d 25 °C for 21 d	*Bacillus cereus*	Bacteriostatic effect	[107]
	Nisin-containing fermentate from *L. lactis*	RTE sliced ham	4 °C for 10 d	*Listeria monocytogenes*	3 log CFU/g	[108]
	5–6 ppm with cinnamaldehyde (15–20 ppm) and grapefruit seed extract (6–8 ppm)	Raw pork loin	4 °C for 12 h	*Listeria monocytogenes*	3 log CFU/g	[71]
Pediocin	5000 bacteriocin units/mL of the pediocin PA-1	Frankfurter	4 °C for 60 d 15 °C for 30 d	*Listeria monocytogenes*	2 log CFU/g 0.6 log CFU/g	[109]
	5000 bacteriocin units/mL of the pediocin PA-1	Frankfurter	10 °C for 60 d 15 °C for 30 d	*Clostridium perfringens*	2 log CFU/g 0.8 log CFU/g	[109]
	Inoculation of pediocin-producing *P. pentosaceus*	Spanish dry-fermented sausages	4 °C for 30 d	*Listeria monocytogenes*	2 log CFU/g	[109]
	320 AU/g with high pressure processing	Portuguese fermented meat sausage	4 °C for 3 d	*Listeria innocua*	2 log CFU/g	[110]
	0.83% with 10% *Murraya koenigii* berries extract	Raw goat meat emulsion	4 °C for 9 d	*Listeria innocua* Total viable counts Psychrophilic count	4.1 log CFU/g 2.2 log CFU/g 1.6 log CFU/g	[111]
Sakacin	Inoculation of sakacin producing *L. sakei*	Beef	5 °C for 14 d	*Listeria monocytogenes*	2 log CFU/g	[112]
	Inoculation of sakacin producing *L. curvatus*	Beef	5 °C for 7 d	*Listeria monocytogenes*	Bactericidal effect	[112]
	3200 AU/mL cell-free supernatant of *L. curvatus*	Meat surface	4–5 °C for 14 d	*Listeria innocua*	Bactericidal effect	[113]
Bacteriocin-like inhibitory substance	Innoculation of BLIS producing *Pediococcus pentosaceus*	RTE pork ham	4 °C for 2 d	* Listeria seeligeri *	0.74 log CFU/g	[114]
	5% of fermented plant using BLIS producing strains	RTC minced pork	18 °C for 12 h	*Escherichia coli* *Enterococcus faecalis* *Staphylococcus aureus* *Streptococcus* spp.	5.53 log CFU/g 4.37 log CFU/g 4.86 log CFU/g 3.84 log CFU/g	[115]
	5 mg/mL of BLIS obtained from *E. faecium*	Chicken surface	4 °C for 72 h	*Clostridium perfringens*	1 log CFU/g	[116]
Mytichitin-CB	6 mg/L of mytichitin-CB peptide expressed by *Pichia pastorisi*	Pork	4 °C for 5 d 4 °C for 8 d 4 °C for 8 d	Total viable counts *Staphylococcus* spp. *Escherichia coli*	1–2 log CFU/g 1–2 log CFU/g 1–2 log CFU/g	[117]
Hispidalin	100 μg/mL of hispidalin expressed by *P. pastorisi*	Pork	4 °C for 7 d	Total viable counts	1 log CFU/g	[118]
